# Isolation of Mal d 1 and Api g 1 - specific recombinant antibodies from mouse IgG Fab fragment libraries – Mal d 1-specific antibody exhibits cross-reactivity against Bet v 1

**DOI:** 10.1186/s12896-015-0157-5

**Published:** 2015-05-27

**Authors:** Jaana Haka, Merja H. Niemi, Kristiina Iljin, Vanga Siva Reddy, Kristiina Takkinen, Marja-Leena Laukkanen

**Affiliations:** VTT Technical Research Centre of Finland Ltd, P.O. Box 1000, Espoo, FI-02044 VTT Finland; Department of Chemistry, University of Eastern Finland, Joensuu Campus, P.O. Box 111, Joensuu, FI-80101 Finland; International Centre for Genetic Engineering and Biotechnology, Aruna Asaf Ali Marg, New Delhi, 110067 India

## Abstract

**Background:**

Around 3–5% of the population suffer from IgE-mediated food allergies in Western countries and the number of food-allergenic people is increasing. Individuals with certain pollen allergies may also suffer from a sensitisation to proteins in the food products. As an example a person sensitised to the major birch pollen allergen, Bet v 1, is often sensitised to its homologues, such as the major allergens of apple, Mal d 1, and celery, Api g 1, as well. Development of tools for the reliable, sensitive and quick detection of allergens present in various food products is essential for allergic persons to prevent the consumption of substances causing mild and even life-threatening immune responses. The use of monoclonal antibodies would ensure the specific detection of the harmful food content for a sensitised person.

**Methods:**

Mouse IgG antibody libraries were constructed from immunised mice and specific recombinant antibodies for Mal d 1 and Api g 1 were isolated from the libraries by phage display. More detailed characterisation of the resulting antibodies was carried out using ELISA, SPR experiments and immunoprecipitation assays.

**Results:**

The allergen-specific Fab fragments exhibited high affinity towards the target recombinant allergens. Furthermore, the Fab fragments also recognised native allergens from natural sources. Interestingly, isolated Mal d 1-specific antibody bound also to Bet v 1, the main allergen eliciting the cross-reactivity syndrome between the birch pollen and apple. Despite the similarities in Api g 1 and Bet v 1 tertiary structures, the isolated Api g 1-specific antibodies showed no cross-reactivity to Bet v 1.

**Conclusions:**

Here, high-affinity allergen-specific recombinant antibodies were isolated with interesting binding properties. With further development, these antibodies can be utilised as tools for the specific and reliable detection of allergens from different consumable products. This study gives new preliminary insights to elucidate the mechanism behind the pollen-food syndrome and to study the IgG epitope of the allergens.

## Background

Allergy is an immunological hypersensitivity disorder to substances in food, air or medical and consumer products, which are normally harmless. At least 30% of the population suffer from IgE-mediated allergic reactions and around 3–5% of them suffer from IgE-mediated food allergies in westernised countries and the number of allergenic people is dramatically increasing [1, 2]. One of the main elicitor of type I allergic reactions worldwide is birch pollen and more precisely one of its major allergens, Bet v 1 [3, 4]. Bet v 1, a 17.4-kDa protein belonging to pathogenesis-related plant proteins (PR-10), is responsible for over 95% of the allergies to birch pollen [5, 6]. Interestingly, over 70% of persons sensitised to birch pollen allergens display adverse reactions to fruits and vegetables as well [7]. Moreover, fresh apple is the most frequently reported food ingredient causing adverse reactions among birch-pollen sensitised individuals [2, 5, 8].

The major apple allergen, Mal d 1, is a 17.5-kDa protein and the member of the same pathogenesis-related protein family that includes Bet v 1 [9]. Mal d 1 and Bet v 1 share approximately 65% amino acid sequence identity [9]. In addition to apple, celery is one of the most important plant food allergen associated with birch pollen sensitisation especially in European countries. It is able to trigger a wide range of allergic symptoms varying from mild oral responses to life-threatening anaphylaxis [2, 10, 11]. The most important allergen in celery tuber is a 16.2-kDa protein Api g 1, also a member of the pathogenesis-related protein family [12]. Api g 1 is a Bet v 1 -homologous protein with approximately 40% amino acid sequence identity with both Bet v 1 and Mal d 1 [12-14].

Interestingly, all of the 1500 allergens identified today are classified to belong to only 2% of known protein families with implications to similar structural and functional characteristics. Furthermore, high homology in the amino acid sequence of the proteins within the same protein family results in homologous secondary and tertiary structures and hence common epitopes of homologous proteins, such as Mal d 1, Api g 1 and Bet v 1. It is surprising, that high homology in protein primary, secondary or even tertiary structure does not necessarily convert into IgE epitope cross-reactivity [15].

Several studies have been conducted in order to explain the cross-reactivity between Bet v 1 and its homologous food allergens, such as Mal d 1 and Api g 1. They are mainly based on molecular modelling, cross-reactive IgE epitope mapping, epitope grafting [16, 17], site-directed mutagenesis [18, 19] or overlapping peptides representing allergens [20]. The crystal structure for the Bet v 1 - IgG Fab fragment BV16 complex has been solved [21] but only a limited amount of information is available on specific antibodies for Mal d 1 or Api g 1. Few monoclonal antibodies have been raised against Mal d 1 using hybridoma technology and their binding to Bet v 1 has been studied, but only a few of these antibodies cross-reacted with Bet v 1 when characterised by immunoblotting and ELISA [22, 23].

The solved allergen-antibody crystal structures strongly imply the importance of native allergen conformation in antibody binding experiments [21, 24-29]. Phage display technology and the availability of large and diverse antibody libraries enable the isolation of allergen-specific recombinant antibodies in a conformation-dependent manner *in vitro* [30, 31]. The bacterial production of functional antibody fragments and their efficient display on a filamentous phage particles have made it possible to construct large and diverse combinatorial antibody phage display libraries for the isolation and evolution of antibodies with an enormous diversity in binding specificities and affinities [32]. In phage display technology, an active antigen/allergen-binding antibody fragment (scFv or Fab fragment) can be displayed on the surface of the phage particle containing the DNA encoding the particular antibody and, thus, the phenotype is linked to the genotype. By this method it is possible to identify antibody phage particles binding specifically to the desired antigen/allergen. Nowadays, several allergen-specific IgE and IgG antibody display libraries have been constructed and recombinant antibodies isolated e.g. against pollen, cow’s milk and latex allergens [30, 31, 33-36].

Here we have isolated IgG antibodies specific for major allergens in apple, Mal d 1 and celery, Api g 1 by the construction and selection of mouse IgG Fab fragment phage display libraries. Recombinant allergens, rMal d 1 and rApi g 1 were expressed and validated prior to mouse immunisations. High-affinity allergen-specific antibodies to rMal d 1 and rApi g 1 were generated using phage display technology. The binding properties and kinetics of the isolated allergen-specific antibodies were characterised utilising the recombinant allergens as well as native allergens from natural sources. The binding properties of the resulting antibodies were also studied against their known homologue, Bet v 1. Interestingly, one of the isolated rMal d 1-specific antibodies bound also to major birch pollen allergen rBet v 1, the main allergen eliciting the cross-reactivity syndrome between the birch pollen and apple. Despite the similarities in Api g 1 and Bet v 1 tertiary structures, the isolated antibodies against rApi g 1 showed no cross-reactivity to rBet v 1 at least within the selected antibodies. These isolated allergen-specific antibodies can be utilised as diagnostic and research tools for detecting allergens and studying allergy-antibody interactions.

## Results

### Characterisation of the recombinant Mal d 1 and Api g 1

**Fig. 1:**
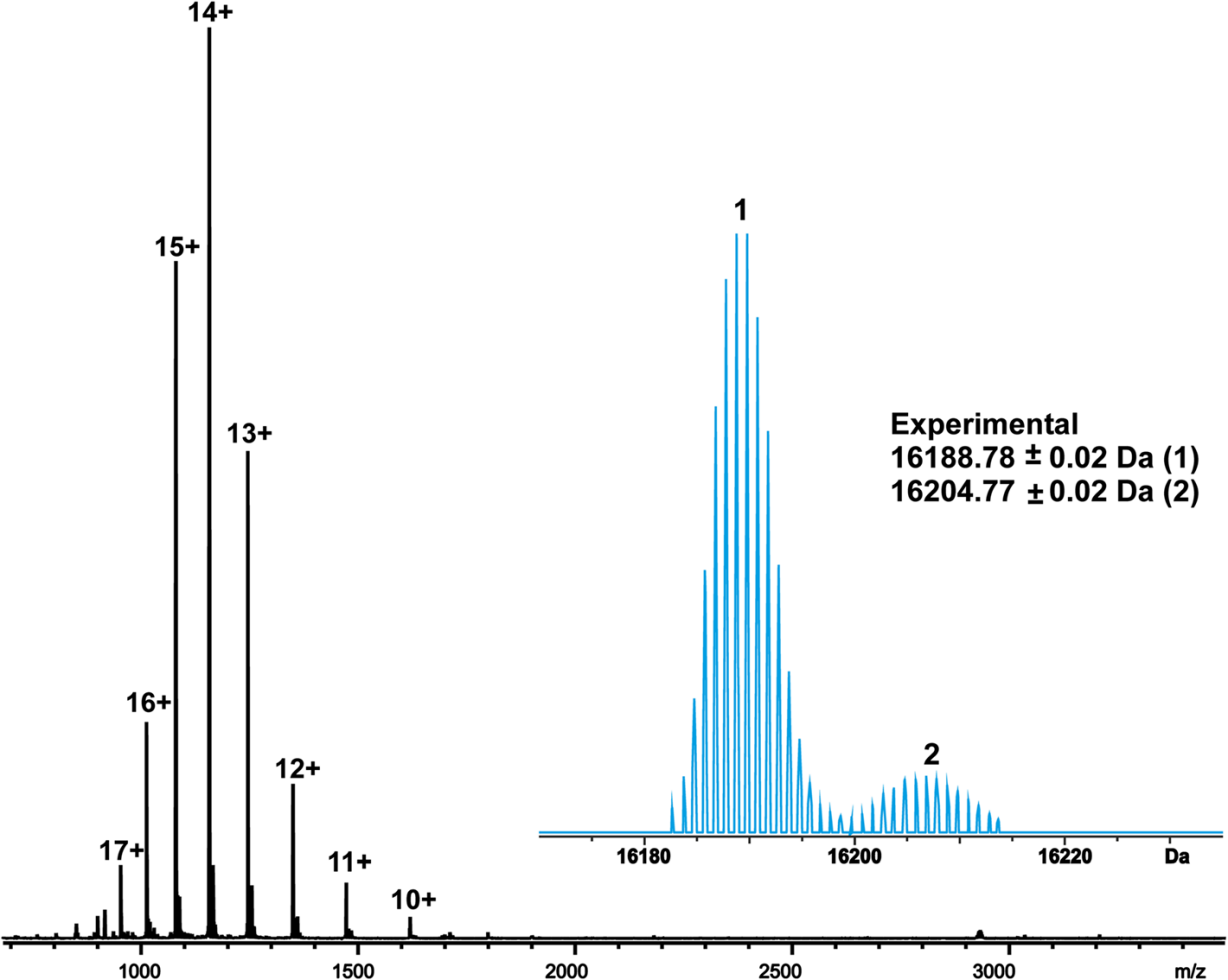
ESI FT-ICR mass spectrum of rApi g 1 measured in MeCN:H_2_O: HOAc (49.5:49.5:1.0, v/v). Numbers denote different protein ion charge states. The deconvoluted mass spectrum has been presented in blue at the right side of the charge-state spectrum. Two protein forms observed in mass spectrum present the full-length (1) and oxidized rApi g 1 (2)

Recombinant allergens, rMal d 1 and rApi g 1 were expressed without any tags or fusions and purified by a two-step chromatography method. The yield from the 1.8-l bacterial culture was 6 mg for rMal d 1 and 17 mg for rApi g 1. According to the analysis of a Coomassie-stained SDS-PAGE gel, the recombinant allergens were purified to a substantial homogeneity (data not shown). In order to further confirm the purity and homogeneity of the purified rApi g 1 and rMal d 1, the accurate molecular masses of the allergens were determined using an ultrahigh-resolution ESI FT-ICR mass spectrometry in denaturing solution conditions. The experimentally determined molecular mass of rApi g 1 (averaged over the charge state distribution) was 16188.78 ± 0.02 Da, which agrees well with the theoretical mass calculated from the full amino acid sequence of Api g 1 (16188.48 Da). The mass spectrum also revealed a partial oxidation of the protein, most likely in a single methionine residue of rApi g 1 (16204.77 Da, Fig. 1).

**Fig. 2:**
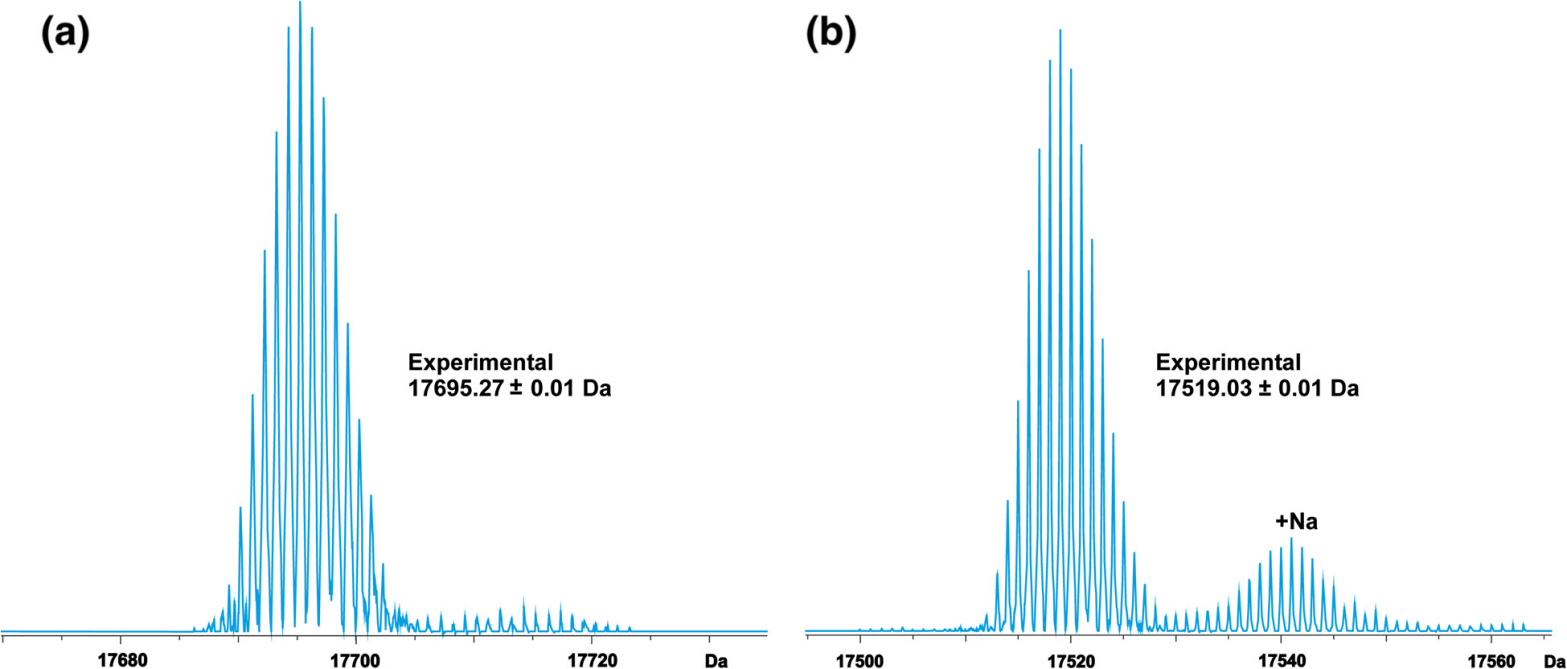
Deconvoluted ESI FT-ICR mass spectra of rMal d 1 measured in MeCN:H_2_O: HOAc (49.5:49.5:1.0, v/v). The deconvoluted mass spectrum has been presented before (**a**) and after (**b**) DTT-reduction

On the contrary, the experimentally determined molecular mass of rMal d 1 (17695.27 ± 0.01 Da, Fig. 2a) was higher than the expected mass of the mature allergen (17518.97 Da). The difference between the experimental and theoretical masses was 176.30 Da. To find out a reason for the observed mass difference, rMal d 1 was enzymatically digested with trypsin. The peptides observed in a mass spectrum of a tryptic digest of rMal d 1 covered 77% of the amino acid sequence of the protein (10 specific tryptic peptides were found, data not shown). Tryptic peptides were not found in the areas spanning the amino acids residues 19–40 and 105–115, thus indicating the location of the possible modification. Interestingly, the latter sequence area of rMal d 1 contained an unpaired cysteine residue C107. Free thiol groups of non-disulfide bridged cysteine residues might be highly reactive and, thus, participate in several electrophilic and oxidative modifications [37]. One of the commonly observed modifications in free cysteine-containing human plasma proteins is the S-cysteinylglycinylation (S-CysGly), which results in a mass increment of +176 Da per each cysteine residue [38, 39]. To analyse whether the observed mass difference in rMal d 1 was caused by the S-CysGly modification, the protein was incubated with a reducing agent. The mass spectrum measured after reduction revealed that the +176.30 Da modification disappeared and the molecular mass of the allergen (17519.03 ± 0.01 Da) corresponded with the theoretical mass (Fig. 2b). The cysteinylglycine dipeptide is also a metabolite of *E. coli*, which explains the finding of S-CysGly modification in a recombinant protein. When S-CysGly was included as the variable modification in the peptide fingerprinting, one additional tryptic peptide was found (residues 98–114), which contains an S-CysGly modified cysteine residue (C107). Hence, the final sequence coverage increased to 84%.

Both rApi g 1 and rMal d 1 contain a single cysteine residue. In the crystal structure of rApi g 1 (PDB:2BK0, [14]), the cysteine residue C114 is buried in the interior of the folded protein. Although the three-dimensional structure of rMal d 1 is not known, the sequence comparison and the mass spectrometric analyses indicate that the C107 is located on the solvent-exposed loop region and, thus, being highly susceptible for oxidative modifications. rMal d 1 and rApi g 1 were used for subsequent mouse immunisations, selections as well as for the screening and characterisation of the allergen-specific Fab fragments.

### Construction and characterisation of the mouse IgG Fab fragment libraries

**Fig. 3:**
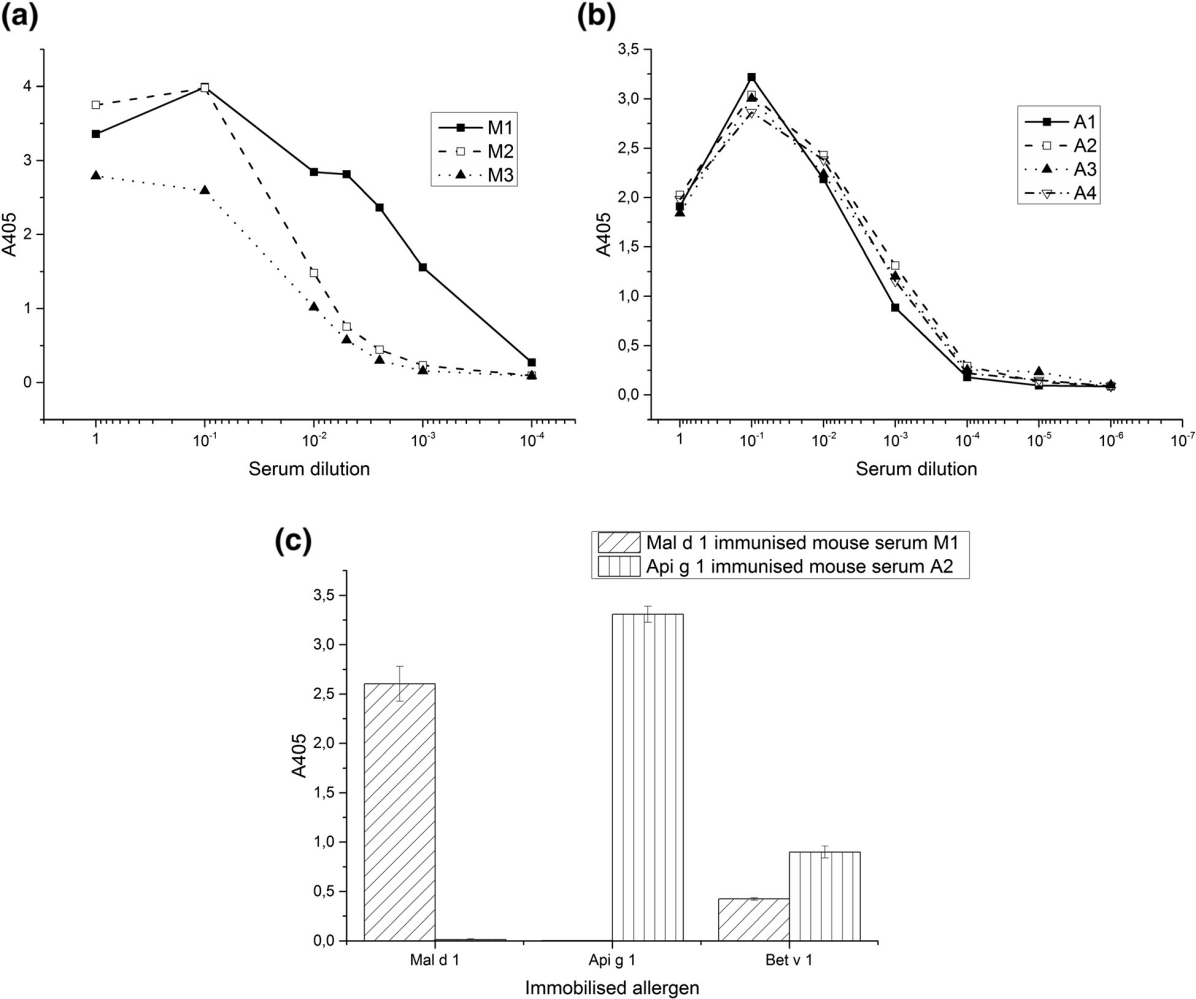
Serum IgG responses of the immunised mice analysed by ELISA. The binding of the mouse serum IgGs to immobilised rMal d 1 (M1–M3) (**a**), rApi g 1 (A1–A4) (**b**) has been analysed. Serum IgG responses of the immunised mice M1 and A2 has also been analysed against the potential cross-reactive allergens, rMal d 1, rApi g 1 and rBet v 1 (**c**). Dilutions of the rMal d 1 (1:200) and rApi g 1 (1:1000) immunised mouse serum samples have been selected according to the Figs. 3a and b, respectively. The results present the mean values of the duplicate samples (±SD)

In order to select the immunised mouse with the highest IgG antibody response against the target allergens, the sera from the immunised mice were tested (Figs. 3a and b). According to the ELISA result, all of the analysed sera expressed considerable IgG immune responses against the target allergens. Based on these results, mouse 1 (M1) and mouse 2 (A2) were selected for the construction of anti-rMal d 1 and anti-rApi g 1 Fab fragment library, respectively. Serum of mouse M1 and A2 was also analysed against potentially cross-reactive allergens, rMal d 1, rApi g 1 and rBet v 1 (Fig. 3c). The results show binding of both rMal d 1 and rApi g 1 immunised mouse sera IgG against rBet v 1. However, rMal d 1 immunised mouse serum IgG showed no binding against rApi g 1 and rApi g 1 immunised mouse serum did not bind to rMal d 1.

Transformation of anti-rMal d 1 and anti-rApi g 1 Fab fragment libraries into *E. coli* XL1-Blue cells resulted in mouse IgG Fab fragment libraries with approximately 10^7^ and 10^5^ independent clones for anti-rMal d 1 and anti-rApi g 1 Fab fragment library, respectively. According to the DNA sequences of the individual unselected Fab fragment clones, all analysed DNA sequences were different in relation to each other (data not shown).

### Isolation of allergen-specific mouse IgG Fab fragments

The selection of the rMal d 1 and rApi g 1 -specific antibody phage libraries was carried out in solution using biotinylated rMal d 1 and rApi g 1, respectively. To follow the enrichment of the selected clones, the ratio between positive (i.e. allergen) versus negative (i.e. BSA) selection were calculated after each selection round. The enrichment was more efficient during anti-rMal d 1 than anti-rApi g 1 antibody selection (data not shown). After the second selection round, the phage pool ELISA was performed for the unselected Fab fragment phage library and for the both enriched Fab fragment phage libraries. According to the rMal d 1 and rApi g 1 -specific Fab fragment phage library ELISA, a clear enrichment between the selection rounds was observed (data not shown).

**Fig. 4:**
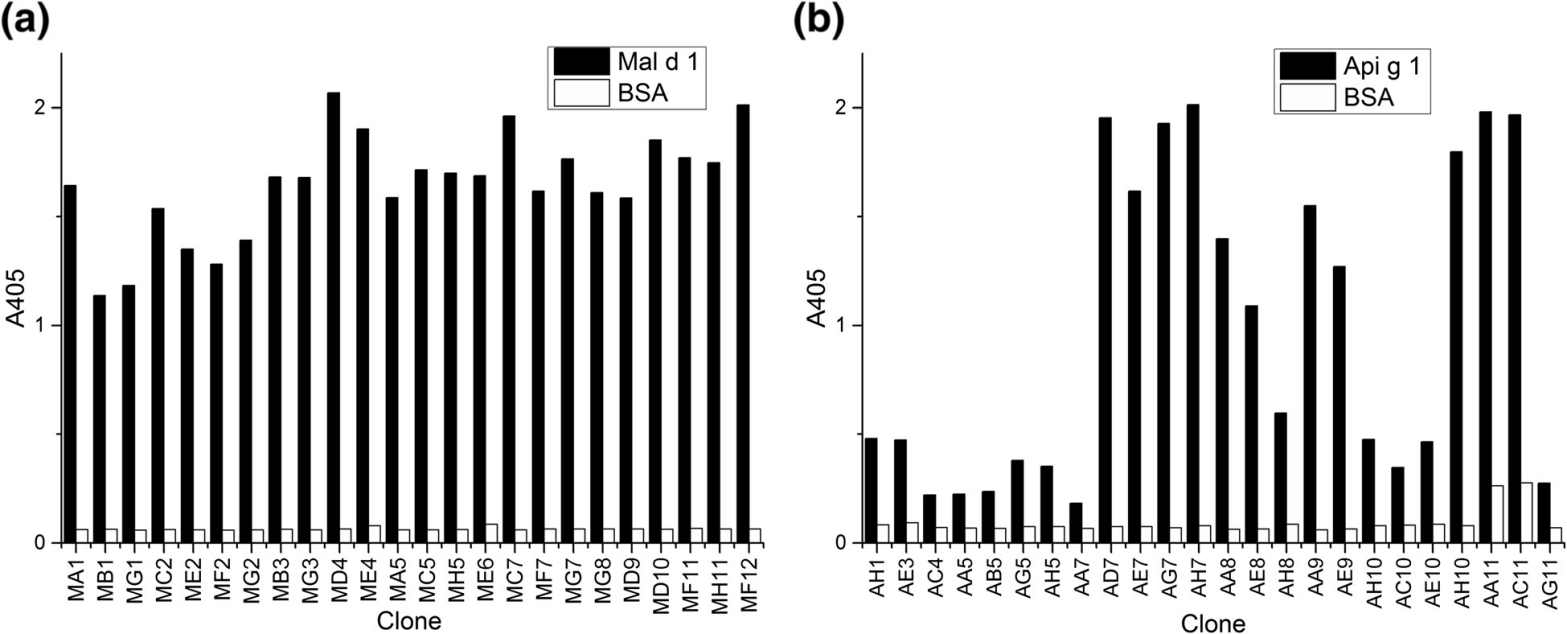
Screening of the rMal d 1 and rApi g 1 -specific antibody phages by ELISA. The specific binding of the individual anti-rMal d 1 (**a**) and anti-rApi g 1 (**b**) Fab fragment phage clones against their target allergen and BSA background has been analysed

Due to observed enrichment in phage pools, individual rMal d 1 and rApi g 1 -specific Fab fragment phage clones were prepared and subjected to ELISA. Total of 96 individual clones from the first and the second selection round were screened for the rMal d 1 and rApi g 1 -specific individual clones by a preliminary ELISA (data not shown). For rMal d 1-specific binders, 35% of the analysed clones from the first selection round and 81% from the second selection round bound to the target allergen. For rApi g 1 binders, the percentages were 13% for the first and 33% for the second selection round. The total of 24 individual rMal d 1 and rApi g 1 -specific antibody phage clones were subjected to a secondary ELISA and screened parallel with BSA background (Fig. 4). In the secondary ELISA, all of the individual phage clones showed binding to the target allergen.

Based on the target allergen binding, the total of ten most promising clones of both rMal d 1 and rApi g 1 -specific antibodies were subjected to DNA sequencing. The results showed a clear similarity between the DNA sequences of each allergen-specific Fab fragments. All other clones were different except two anti-rApi g 1 Fab fragment clones. There were two identical sequences (AA11 and AC11) out of ten clones. Two Fab fragment clones per allergen, designated as MA1 and MB3 for rMal d 1-specific and AH7 and AA11 for rApi g 1-specific, were chosen for further characterisation. The variations in the sequences and binding profiles of the Fab fragments were used as the selection criteria. The complementarity-determining regions (CDRs) of the selected anti-rMal d 1 and anti-rApi g 1 Fab fragments were determined and are shown in Table 1. Similarity of the amino acid sequences between the two allergen-specific Fab fragments MA1 and MB3 as well as AH7 and AA11 is expectedly seen, with a minimum of one amino acid difference in the CDRs. For rApi g 1-specific Fab fragment clones, CDRs for heavy framework 1 and 3 (HCDR1 an HCDR3, respectively) and light framework 2 (LCDR2) are identical.

**Table 1:** CDRs of the allergen-specific Fab fragments

Fab	HCDR1	HCDR2	HCDR3	LCDR1	LCDR2	LCDR3
MA1	GFTFS**A**YGMS	TIS**N**GG**T**YTYY**Q**DSVKG	PPS**R**GGYFDV	RAS**E**NI**YS**NLA	AAK**T**LAD	QHFW**S**TP**F**T
MB3	GFTFS**N**YGMS	TIS**S**GG**S**YTYY**P**DSVKG	PPS**K**GGYFDV	RAS**G**NI**HN**NLA	AAT**N**LAD	QHFW**D**TP**W**T
AH7	GFDFSRYWMS	EINPDSSTINY**T**PSLKD	FPMDY	KASQ**D**V**STA**VV	SASYRYS	QQYN**N**YPYT
AA11	GFDFSRYWMS	EINPDSSTINY**P**PSLKD	FPMDY	KASQ**N**V**GIN**VV	SASYRYS	QQYN**S**YPYT

Differences between the same allergen-binding Fab fragments are indicated in bold.

### Characterisation of binding properties of the allergen-specific mouse IgG Fab fragments

**Fig. 5:**
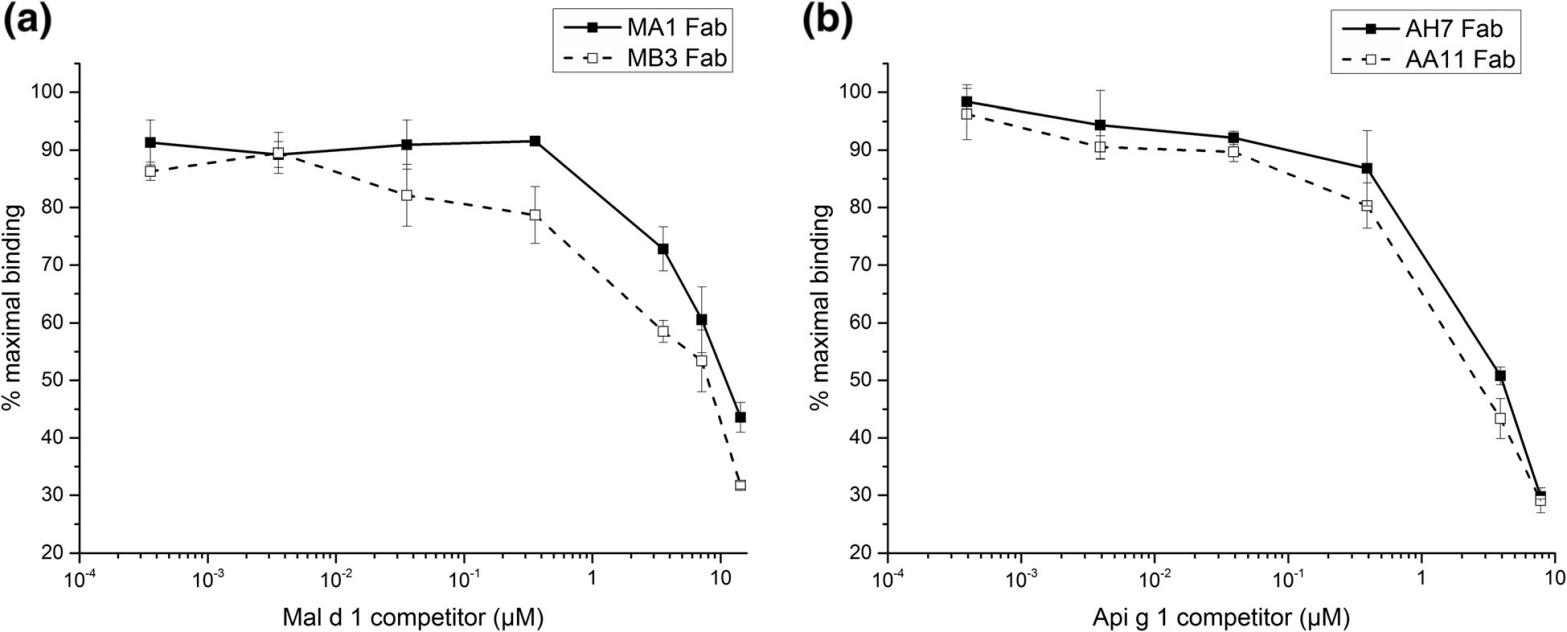
Binding properties of the Fab fragments analysed by competitive ELISA. The binding properties of the purified anti-rMal d 1 (**a**) and anti-rApi g 1 (**b**) Fab fragments have been analysed. The results present the mean values of the duplicate samples (±SD)

To further characterise the allergen-binding properties of the MA1 and MB3 as well as AH7 and AA11 Fab fragment, they were produced in a larger scale as soluble Fab fragments containing hexahistidine tag and, thus, purified from the culture supernatant by IMAC. The purification yield of the Fab fragments from 900 ml of culture supernatant was approximately 1 mg for all of the Fab fragments, except for the MA1 Fab fragment with 7.8 mg. After the purification, the Fab fragments were subjected to the competitive ELISA to assess their binding specificity against their target allergens in more detail (Fig. 5). The Fab fragments showed specific binding to the target allergens as their binding to the target allergens was displaced in a concentration-dependent manner by a competing allergen.

The binding affinities and kinetics were further analysed by Surface Plasmon Resonance (SPR) experiments (Table 2). Based on the measured affinity constants (K_D_), the MA1 and MB3 Fab fragments showed higher affinity than the AA11 and AH7 Fab fragments to the target allergens. The MA1 Fab fragment bound rMal d 1 with the higher affinity compared to MB3 Fab fragment (K_D_ of 3.3 nM and 19.7 nM, respectively). Furthermore, the AH7 Fab fragment bound rApi g 1 with slightly higher affinity compared to AA11 Fab fragment (K_D_ of 0.54 μM and 0.70 μM, respectively).

**Table 2:** The kinetic constants of allergen-specific Fab fragments measured by BIAcore

Allergen	Fab	k_a_ (1/Ms)	k_d_ (1/s)	K_D_ (M)
rMal d 1	MA1	7.340 × 10^5^ ± 1.3 × 10^3^	0.002427 ± 2.1 × 10^−6^	3.306 × 10^−9^
rMal d 1	MB3	2.990 × 10^5^ ± 1.6 × 10^3^	0.005890 ± 2.1 × 10^−5^	1.970 × 10^−8^
rApi g 1	AH7	8.056 × 10^4^ ± 1.6 × 10^3^	0.04323 ± 7.6 × 10^−4^	5.366 × 10^−7^
rApi g 1	AA11	8.533 × 10^4^ ± 5.1 × 10^2^	0.05931 ± 2.1 × 10^−4^	6.950 × 10^−7^

The association (k_a_) and dissociation (k_d_) rate constants and affinity constant (K_D_) have been measured for the Fab fragments. The values present the averages (±SD) obtained with seven different Fab fragment concentrations.

**Fig. 6:**
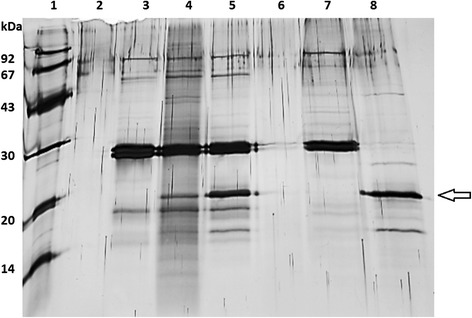
Binding of the Fab fragment to the native allergen analysed by immunoprecipitation assay. An apple extract precipitated with MA1 Fab fragment is shown here. A silver-stained SDS-PAGE gel with low molecular weight marker (lane 1), only beads (lane 2), beads incubated with Fab fragment (lane 3), beads incubated with Fab fragment and allergen extract containing the native form of Mal d 1 (lane 4), beads incubated with Fab fragment and purified rMal d 1 (Biomay, lane 5), beads incubated with allergen extract containing the native form of Mal d 1 (lane 6), Fab fragment control (lane 7) and purified rMal d 1 control (Biomay, lane 8). The arrow is showing Mal d 1

In ELISA and SPR experiments, the analysis of the binding properties of the Fab fragments was carried out using purified, recombinant allergens. Next, the ability of Fab fragments to recognise the native allergen extracted from a natural source was analysed using immunoprecipitation assay. All of the allergen-specific Fab fragments precipitated their target native allergen as well as purified recombinant allergen (Fig. 6).

**Fig. 7:**
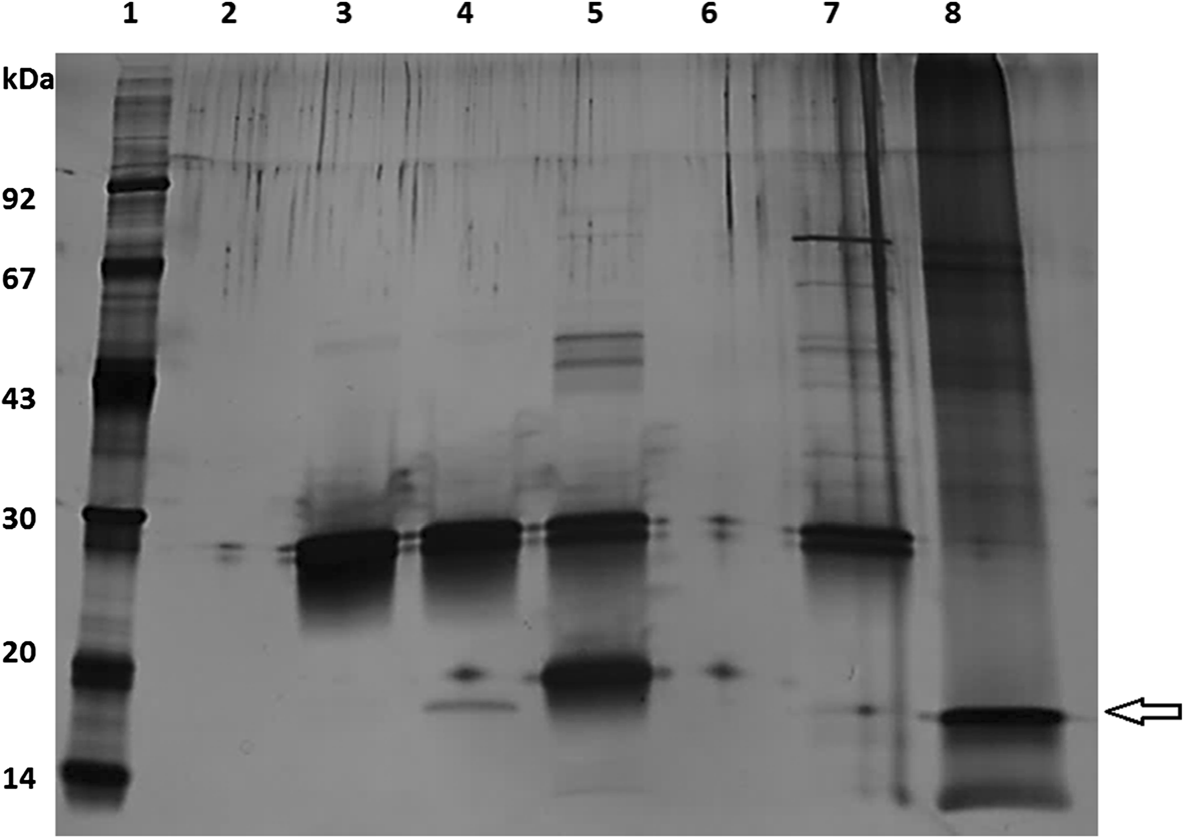
Binding of the MA1 Fab fragment to rBet v 1 measured by immunoprecipitation assay. A silver-stained SDS-PAGE gel with low molecular weight marker (lane 1), only beads (lane 2), beads incubated with Fab fragment (lane 3), beads incubated with Fab fragment and rBet v 1 (Biomay, lane 4), beads incubated with Fab fragment and rMal d 1 (Biomay, lane 5), beads incubated with rBet v 1 (lane 6), Fab fragment control (lane 7) and rBet v 1 control (Biomay, lane 8). The arrow is showing rBet v 1

Due to Mal d 1, Api g 1 and Bet v 1 amino acid sequence similarity, structural similarity of Api g 1 and Bet v 1 allergens [14, 40] as well as the observed cross-reactivity of the immunised mice sera (Fig. 3c), the cross-reactivity of the Fab fragments against the rBet v 1 was analysed by ELISA (data not shown), immunoprecipitation assays (Fig. 7) and SPR experiments (Table 3). The Fab fragment in the culture supernatant or as purified form were allowed to bind rMal d 1, rBet v 1, rApi g 1 and BSA. Only the anti-rMal d 1 Fab fragment clone MA1 showed cross-reactivity against rBet v 1. The result was confirmed using purified Fab fragments in ELISA and SPR experiments. The MA1 Fab fragment bound rBet v 1 with lower affinity than rMal d 1 (K_D_ of 29.7 nM and 3.3 nM, respectively). There were no observable binding of anti-rApi g 1 Fab fragments or anti-rMal d 1 Fab fragment MB3 against any other than their target allergen.

**Table 3:** The kinetic constants of MA1 Fab fragment against rBet v 1

Allergen	Fab	k_a_ (1/Ms)	k_d_ (1/s)	K_D_ (M)
rBet v 1	MA1	1.986 × 10^5^ ± 2.8 x 10^3^	0.005705 ± 5.2 x 10^−5^	2.972 × 10^−8^

The association (k_a_) and dissociation (k_d_) rate constants and affinity constant (K_D_) have been measured for MA1 Fab fragment against rBet v 1. The values present the averages (±SD) obtained with seven different Fab fragment concentrations.

**Fig. 8:**
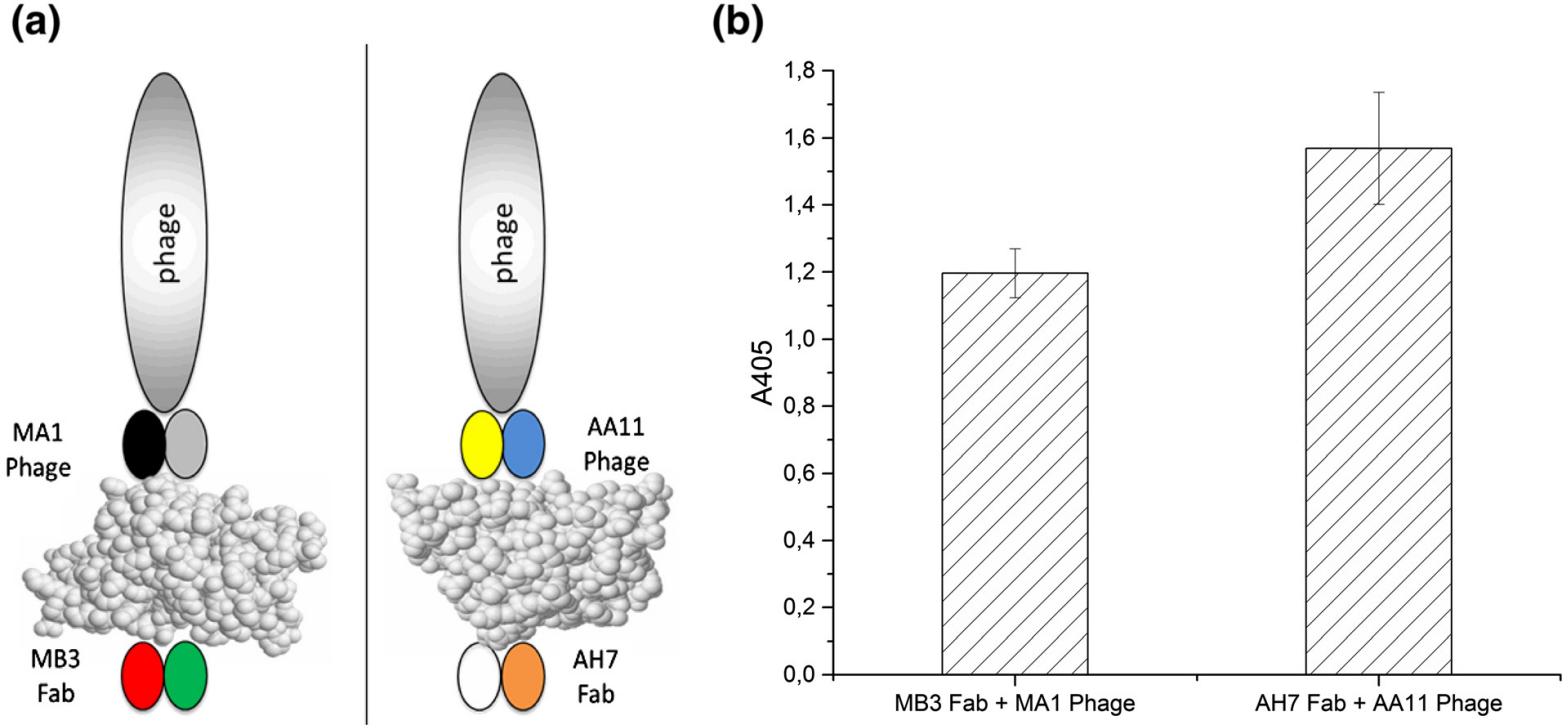
Sandwich ELISA. The sandwich ELISA principle (**a**) and result (**b**) are shown to one sandwich couple per allergen. The immobilised antibody is the purified Fab fragment (MB3 or AH7 Fab fragment) whereas the detection antibody is displayed on the tip of the phage particle (MA1 or AA11 Fab fragment displaying phage). The front view of Bet v 1 (left, PDB:1BV1, [40]) and Api g 1 (right, PDB:2BK0, [14]) crystal structures are from Protein Data Bank. Since Mal d 1 crystal structure is not accessible, the structure of its homologue Bet v 1 was used here as a model of Mal d 1 allergen

The usability of allergen-specific Fab fragments for food allergen detection was studied by sandwich ELISA (Fig. 8). The sandwich ELISA proved to be functional with both of the combinations of allergen-specific Fab fragment and antibody-phage fusion. The result of the sandwich ELISA for rMal d 1 and rApi g 1 are shown in Fig. 8b.

## Discussion

Combinatorial antibody phage display technology has proven to be highly powerful technique for the isolation of target-specific IgE and IgG antibodies for a wide variety of molecules [30, 31, 33-36]. Here, we were able to isolate several unique and specific recombinant Fab fragments exhibiting high affinity against recombinant and native Mal d 1 and Api g 1, the major apple and celery allergen, respectively. To our best knowledge, this is the first study reporting the isolation of monoclonal antibodies against Api g 1 and the second study after Fahlbusch *et al*. [23] reporting isolation of monoclonal antibodies against Mal d 1. We characterised the binding properties of the isolated recombinant antibodies in more detail compared to the earlier published studies of Mal d 1 antibodies including ELISA, SPR experiments and immunoprecipitation assays. The allergen-specific Fab fragments bound to their target allergen with high affinity (10^−7^–10^−9^ M). These results correlate with the affinities of human IgE antibodies against β-lactoglobulin [30] and hevein (unpublished, Laukkanen *et al.*) isolated using a similar phage display approach. The rMal d 1-specific Fab fragments are higher in their affinities (10^−8^–10^−9^ M) against their target allergen compared to rApi g 1-specific Fab fragments (10^−7^ M). Both rApi g 1-specific Fab fragments AH7 and AA11 behave similarly when studied in competitive ELISA and SPR experiments. Interestingly, although the differences between the isolated rMal d 1-specific Fab fragments MA1 and MB3 in the amino acid sequence level are minor (Table 1), their influence to the allergen binding properties is significant. While the allergen-binding properties of MA1 and MB3 Fab fragments are similar according to the rate constants, there is 10-fold difference in their affinity constants (Table 2). The MA1 and MB3 Fab fragments have minor differences in all of their CDRs, including HCDR3 which is especially important for antibody specificity [41]. The AH7 and AA11 Fab fragments, on the other hand, have identical HCDR3 as well as HCDR1 and LCDR2, which may explain similar binding characteristics.

Allergens of the Bet v 1 protein family, such as major allergens in apple, Mal d 1, and in celery, Api g 1, are the main cause of pollen-related food allergies after the first sensitisation to birch pollen. Moreover, persons sensitised to Bet v 1 are often sensitised to its homologues, such as Mal d 1 and Api g 1 as well. Despite the globally observed adverse connection among birch pollen sensitised individuals to certain food ingredients, such as apple and celery, there remain a lack of published data concerning the molecular mechanism of the cross-reactivity. Antibodies are great tools for deepening the understanding of molecular interactions initiating an allergic response. Hecker *et al.* [16] developed the first Bet v 1-specific human IgE scFv fragment containing a V_H_ with human origin and a synthetic V_L_. While studying its cross-reactivity against Mal d 1, they concluded that the absence of cross-reactivity was due to a less accessible Mal d 1 residue E148 compared to that of Bet v 1. According to Levin *et al*. [36] who were able to isolate Bet v 1-specific human monoclonal IgE from the combinatorial antibody fragment library, the antibody did not cross-react with Mal d 1 due to differences occurring in the segment consisting of interactive allergen residues I56-K65. Although allergic reactions are IgE-mediated, it has been recognised that allergen-IgG complex can serve as a model for the study of allergen-IgE interactions [21]. Previously isolated anti-Bet v 1 IgG antibody BV16 [21] is not showing cross-reactivity towards Mal d 1 but to the point mutated form of Mal d 1 exhibiting BV16 epitope [17]. Schirmer *et al*. [14] compared the crystal structures of Bet v 1 and Api g 1, and they concluded that Api g 1 lacks the residue E45 which has been shown to be crucial for IgG antibody BV16 binding against Bet v 1 [21] and, thus, explaining the absence of cross-reactivity. Here we analysed the binding of the anti-rMal d 1 and anti-rApi g 1 Fab fragments against rBet v 1. Based on the binding studies, the rMal d 1-specific MA1 Fab fragment bound also to rBet v 1. The SPR experiment showed that the complex formation occurred with the high affinity (10^−8^ M, Table 3) and indicates that the MA1 Fab fragment association with rBet v 1 requires approximately 3.7-fold more time than association with rMal d 1. According to the results, there are no binding of MB3 Fab fragment against rBet v 1. This further underlines the remarkable effect of the minor differences in the amino acid sequences of the Fab fragments to their binding characteristics. According to the results, rApi g 1-specific Fab fragments exhibit no binding to rBet v 1. These findings are in congruent with previous studies of Fahlbusch *et al*. [23], where only three of the eight studied Mal d 1-specific antibodies showed cross-reactivity against Bet v 1. Also the smaller size of the Fab fragment library (10^5^ clones) constructed from the Api g 1 immunised mouse (A2) compared to the anti-Mal d 1 Fab fragment library (10^7^ clones) resulted in the lower number of the Fd region and light chain combinations and this might explain the lack of Bet v 1 cross-reactive clones. Additionally, the anti-Api g 1 Fab fragments isolated from the antibody library showed around ten-fold lower binding affinity (Table 2) compared to the anti-Mal d 1 Fab fragments and this might have an effect to the result as well. Therefore, even though none of the studied rApi g 1-specific Fab fragments exhibit cross-reactivity against rBet v 1, it is possible to find cross-reactive clones by analysing more clones gained from combinatorial phage display library as rApi g 1 immunised mouse sera IgG showed binding against rBet v 1.

Even the intake of trace amount of food allergens can cause a minor to a fatal allergic reaction to sensitised individuals. Therefore, it is important to have reliable and sensitive methods for the detection of a minute amount of allergens in different food matrices. Traditionally the diagnostics of allergens are based on the use of human IgE serum pools isolated from allergic patients. The use of monoclonal antibodies would ensure the specific detection of the harmful food content for a sensitised person. In addition to consumer-safety issues, there are also international regulations for labelling food containing allergenic ingredients [42]. Allergen-specific IgG Fab fragments can be utilised as tools for allergen detection purposes especially in sandwich ELISA, lateral flow and microarray-based test kits to improve food safety [43]. Here, we have shown by using immunoprecipitation assays and allergen extracts from apple and celeriac for Mal d 1 and Api g 1, respectively, that the isolated Fab fragments recognise also their target allergens extracted from the natural sources. For better detection, the affinity maturation can be used to further improve sensitivities of the Fab fragments if needed [44]. Here, we have also shown by using a sandwich ELISA that these allergen-specific Fab fragments may have great potential in food allergen detection applications in form of ELISA or lateral flow-based assays.

Despite the enhanced potential and effort put in characterising molecular properties of allergens, only a few studies have aimed at understanding the differences between the allergenic IgE and immunogenic IgG epitopes of the allergens. Based on the published antibody-allergen structures, it has been concluded that although IgE and IgG epitopes differ in geometrical structure they share some similarities originating from the producing state of the antibodies [45]. Here we demonstrate that allergen-specific Fab fragments isolated by the conformational manner using antibody phage display technology are useful tools in studies of these allergen-antibody interactions. Future studies include expanding the idea of using phage display library derived allergen-specific antibodies as tools for characterising allergen-antibody structures and epitopes of human antibodies. Human IgE and IgG phage display libraries have been successfully constructed from sensitised persons [e.g. 33, 35] and IgE and IgG antibodies isolated for instance against food allergens [30] and Bet v 1 [33, 36]. Therefore, it is of interest to compare whether similar cross-reactive antibodies can be isolated from the allergen-specific human IgEs. Also, to further compare the antibody-allergen interactions, crystal structures of the antibody-allergen immunocomplexes are needed [29]. Crystal structures may reveal antibody-binding epitopes and therefore, could be used to find out whether MA1 and MB3 Fab fragments recognise different Mal d 1 epitopes.

## Conclusions

In summary, we have isolated and identified in more details a total of four novel, two rMal d 1 and rApi g 1 -specific, Fab fragments with the high affinities from the mouse IgG Fab fragment libraries. We have demonstrated that these allergen-specific antibodies recognise their targets presented either as a recombinant or native protein. The binding ability of antibody to its target food allergen as its native form prevalent in natural sources is important when designing detection methods for tracing allergens from different food matrices. Interestingly, one of the rMal d 1-specific Fab fragments bound also to rBet v 1, hence exhibiting cross-reactivity. Therefore, this study gives new preliminary insights to elucidate the mechanism behind the pollen-food syndrome as well as to study the IgG epitope of the allergens.

## Methods

### Production of recombinant Mal d 1 and Api g 1

Recombinant major apple and celery allergens, rMal d 1 and rApi g 1, respectively, were produced as soluble, non-fusion proteins in bacterial cells using pKKtac expression vector and *Escherichia coli* strain RV308 as described by Takkinen *et al.* [46]. Briefly, expression of recombinant proteins in bacterial cells resulted in the secretion of soluble proteins into the periplasmic space. Allergens were isolated from the periplasm by osmotic shock as described by Boer *et al.* [47]. Purification was performed by hydrophobic interaction chromatography (HIC, Phenyl Sepharose 6 Fast Flow medium, GE Healthcare) with a linear gradient of 20 mM monosodium phosphate buffer containing 1 M NaCl at pH 5.0 and 20 mM Tris buffer containing 7.5% isopropanol at pH 9.3 followed by size exclusion chromatography (HiLoad Superdex 75 Column, GE Healthcare) with PBS as elution buffer. Purified allergens were analysed by Coomassie-stained SDS-PAGE gels and validated by mass spectrometry. As control proteins, commercially available recombinant allergens rMal d 1 (Mal d 1.0108), rApi g 1 and the major birch pollen allergen, rBet v 1 (Bet v 1.0101), were purchased from Biomay.

Mass spectrometric measurements were performed with an APEX-Qe Fourier transform ion cyclotron resonance (FT-ICR) mass spectrometer (Bruker Daltonics, Billerica, MA, USA), equipped with a 12 T superconducting magnet and a Bruker Apollo-II Ion-Funnel ESI source. Before mass spectrometric analyses, protein samples were purified from the buffering salts with PD-10 columns (Amersham-Biosciences), using MS-compatible ammonium acetate buffer (10 mM, pH 6.9) as an eluent. For mass spectrometric measurements in denaturing solution conditions, protein samples were diluted to 2 μM with MeCN:H_2_O: HOAc (49.5:49.5:1.0, v/v) and the solution was introduced by a syringe pump with the flow rate of 1.5 μl/min into the ion source. Nitrogen was used as the drying (250°C, 6 mbar) and nebulizing gas. Ions from the ESI-source were accumulated in the hexapole ion trap for 1.0 s and transferred to the ICR cell for trapping, excitation and detection. Data collection and processing were carried out by using Bruker XMASS software (version 7.0.8). For each mass spectrum, a total of 256 co-added (1 Mword) time-domain transients were recorded and fast Fourier transformed before the magnitude calculation. Mass spectra were calibrated externally with respect to the ions of an ES Tuning Mix (Agilent Technologies).

For trypsin digestion, lyophilized trypsin from bovine pancreas (Sigma Aldrich) was dissolved in 10 mM ammonium bicarbonate buffer (pH 8.2) and mixed with rMal d 1 solution using an enzyme:protein ratio of 1:30 (w/w). The sample was incubated for four hours at 37°C. The digestion was quenched by adding a small amount of formic acid and the digest was frozen at − 20°C. Before mass spectrometric analysis, the sample was diluted to 2 μM with acetonitrile. The mass spectrum was recorded as described earlier. The digestion spectrum was interpreted using DataAnalysis 4.0 software (Bruker Daltonics) and the tryptic peptides were identified using GPMAW software (version 9.22, Lighthouse data).

In order to study the effect of the free cysteine residue to the observed mass difference, the rMal d 1 was reduced by incubating the protein solution with 10 mM dithiothreitol (DTT) at 37°C for three hours. The denatured mass spectrum was recorded immediately after the DTT reduction.

### Construction of the mouse anti-Mal d 1 and anti-Api g 1 IgG Fab fragment libraries

Total of four BALB/c mice per allergen were immunised separately with rMal d 1 and rApi g 1. All the mouse experiments were in accordance with adequate guidelines on animal welfare guided by Institutional Animal Ethics Committee, Committee for the Purpose of Control and Supervision of Experiments on Animals in India and Animal Experiment Board in Finland. Serum IgG responses of three rMal d 1 and four rApi g 1 immunised mice were determined by ELISA. The biotinylated allergen (0.5 μg) was immobilised onto streptavidin (SA) -coated microtiter plate wells (Kaivogen). Dilution series of the serum samples were added to the allergen-coated wells and incubated for an hour at room temperature with agitation. The binding of IgG antibodies to the allergens was detected using AFOS-conjugated goat anti-mouse IgG (H + L) (BioRad) and *p*-nitrophenylphosphate substrate (Sigma Aldrich). The absorbance values were measured at 405 nm (SPECTROstar Omega, BMG Labtech). The binding of IgG fragments from the sera of rMal d 1 and rApi g 1 immunised mice were also analysed against rBet v 1. Serum dilutions of 1:200 and 1:1000 were used for rMal d 1 and rApi g 1 immunised mouse serum, respectively.

Based on the ELISA result, spleens from the mouse with the highest serum IgG responses against rMal d 1 and rApi g 1 were chosen for the Fab fragment library construction. Spleens were homogenised (Polytron PT 1200 Homogenizator, Kinematica) and total RNA was isolated according to the RNeasy Midi Kit protocol (Qiagen). First strand cDNA synthesis was carried out using Transcriptor First Strand cDNA Synthesis Kit (Roche) according to manufacturer’s protocol and using oligo (dT) primer. Mouse kappa light chains and IgG heavy chains were amplified separately from the synthetized cDNA by using primers based on an article from Krebber *et al*. [48]. Briefly, 3’ primers were designed for annealing to the constant coding regions (C_L_ and C_H1_) and a total of 17 and 19 different 5’ primers for the amplification of light and heavy variable domains (V_L_ and V_H_), respectively. The amplification was carried out in a two-step PCR reaction. The first step produced amplified regions that were used as a template for the second PCR reaction. In the second step specific restriction sites were added to the 5’ end of the DNA fragments. The same set of 3’ primers containing specific restriction sites were used for the primary and the secondary amplification reaction. The amplified light and Fd regions were ligated into pJET1.2/blunt cloning vector (Thermo Scientific) as blunt-end fragments and cleaved with specific restriction enzymes to improve cloning efficiency to a final phagemid vector, a display vector. Finally, the amplified and digested DNA fragments encoding light chains and Fd regions were pooled and cloned into the previously described phagemid vector [31]. The resulting mouse anti-rMal d 1 and anti-rApi g 1 Fab fragment libraries were validated by restriction enzyme digestions and sequencing ten independent clones from each library (GATC Biotech).

### Selection of the mouse IgG antibody libraries

Biotinylated allergens were used in the selection of mouse antibody libraries. For biotinylation reaction, recombinant allergens (Biomay) and bovine serum albumin (BSA, Sigma Aldrich) used as a negative background control were diluted in 50 mM sodium bicarbonate buffer, pH 8.5 or PBS. Protein solutions were incubated with 10 mM biotin reagent (EZ-Link Sulfo-NHS-LC-Biotin, Thermo Scientific) for 30 min at room temperature. In the labeling reaction, a 10-fold molar excess of biotin reagent was used. The biotinylation of rMal d 1, rApi g 1, rBet v 1 and BSA (rMal d 1-Bio, rApi g 1-Bio, rBet v 1-Bio and BSA-Bio, respectively) was confirmed by immunoblot using alkaline phosphatase conjugated streptavidin (Amersham Biosciences) as a detection antibody.

The selection of rMal d 1 and rApi g 1-specific antibodies from the constructed Fab fragment libraries was based on the specific protein-protein interactions. For the selection, phage particles displaying Fab fragments on the surfaces of the filamentous M13 bacteriophages were prepared. The resulting phagemid DNA from the Fab fragment library construction was transformed into *E. coli* XL1-Blue electroporation competent cells (Agilent Technologies). The volume of the cultivation was increased with Super Broth (SB) medium supplemented with carbenicillin (20 μg/ml) and tetracycline (10 μg/ml) and incubated for an hour at 37°C with agitation followed by a second supplementation with carbenicillin (50 μg/ml). The phage particles were then assembled using a helper phage (Stratagene, VCS M13, 10^11^ cfu/ml). After incubation for 30 min at 37°C, the volume of the cultivation was again increased to 100 ml with SB medium supplemented with carbenicillin (50 μg/ml) and tetracycline (10 μg/ml). After two hours of incubation at 37°C with the agitation, the cultivation was supplemented with kanamycin (70 μg/ml). After an 18-h incubation step at 34°C with the agitation, the phage particles were purified twice by a precipitation with PEG-6000 (Sigma Aldrich) and 2.5 M NaCl.

During the selection, anti-rMal d 1 and anti-rApi g 1 antibody phage pools (10^10^ cfu/ml) were allowed to react in solution with the biotinylated rMal d 1 (C_final_ = 630 nM) or rApi g 1 (C_final_ = 690 nM), respectively. To control the allergen-specific binding, the antibody phage pools were also allowed to react with biotinylated BSA (C_final_ = 160 nM). After two hours of incubation at room temperature with rotation, the reaction solutions were transferred to the SA-coated microtiter plate wells and incubated for 30 min at room temperature with agitation. After washing step, the specifically bound phages were eluted with 0.1 M Glycine-HCl, pH 2.0 and immediately neutralised with 2 M Tris. For the next selection round, the eluted phage pools were amplified by infecting *E. coli* XL1-Blue cells and phage particles re-assembled with helper phages followed by the phage precipitation as described earlier. Total of two selection rounds were performed.

### Screening of allergen-specific antibodies by ELISA

The preliminary screening of rMal d 1 and rApi g 1 -specific Fab fragments was carried out by ELISA using Fab fragment phage pools. rMal d 1-Bio, rApi g 1-Bio and BSA-Bio (0.5 μg) were immobilised on SA-coated microtiter plate wells as described earlier. Dilution series of the original phage library as well as amplified Fab fragment phage pools after each selection round were added to the allergen-coated wells and incubated for two hours at room temperature with agitation. Specifically bound phage particles were detected using anti-M13-HRP conjugate (GE Healthcare) and 2,2-azino-bis (3-ethyl-benzothiazoline-6-sulfonic acid) di-ammonium salt (ABTS, Roche). The absorbance values were measured at 405 nm.

Based on the phage pool ELISA results, the secondary screening of individual rMal d 1 and rApi g 1 -specific Fab fragment clones was conducted by ELISA. Individual bacterial clones were picked from different selection rounds and grown in Super Broth (SB) medium supplemented with ampicillin (100 μg/ml), tetracycline (10 μg/ml) and 1% glucose in 96-deep well plates for 18 h at 37°C with agitation. The cultivations were then diluted 1:50 in SB medium to a final volume of 1 ml supplemented with ampicillin (100 μg/ml) and tetracycline (10 μg/ml) and grown for three hours at 37°C with agitation. After adding helper phage (10^9^ cfu), the cultures were subjected to a helper phage infection for 30 min at 37°C and a 2-h period of growth at 37°C with agitation. Finally, the cultures were supplemented with kanamycin (70 μg/ml) and cultivated for 18 h at 34°C with agitation. The cells were then harvested by centrifugation (3200 g, 20 min, 4°C). The supernatants containing Fab fragment-pIII fusions were subjected to the ELISA. The immobilisation of the biotinylated allergens and BSA onto SA-coated wells was carried out as previously. Phage particles displaying Fab fragments obtained from the selection rounds were allowed to bind to the allergen for two hours at room temperature with agitation. As previously, the specifically bound phage clones were detected with anti-M13-HRP conjugate and ABTS. The absorbance values were measured at 405 nm. Based on the ELISA results, total of 20 clones, i.e. five clones from each selection round were subjected to DNA sequencing (GATC Biotech). Sequences were aligned with Geneious software (version 6.1.6, Biomatters Ltd) and CDR regions identified.

### Production and purification of allergen-specific Fab fragments

For further characterisation, two mouse anti-rMal d 1 and anti-rApi g 1 IgG Fab fragments were selected according to the sequence analysis. First, the coding sequences of the Fd region and light chain of the Fab fragments were cloned into pKKtac bacterial expression vector containing the sequence encoding the hexahistidine tag at the Fd region C-terminus. The resulting recombinant Fab fragments were produced in *E. coli* RV308 strain as described earlier [46]. From the supernatants, the Fab fragments were purified using standard immobilised metal ion affinity chromatography (IMAC) procedure as described earlier [49]. Briefly, TB (Terrific Broth) medium containing ampicillin (100 μg/ml) and 0.1% glucose was inoculated 1:50 with 18-h pre-culture of Fab fragments, and grown until OD_600_ value of 4 was reached. The Fab fragment expression was induced by adding 1 mM IPTG. The culture medium was supplemented with additional ampicillin (100 μg/ml) and cultivated at 30°C and 170 rpm for 18 h. Finally, the cells were harvested by centrifugation (4500 g, 15 min, 4°C) and the Fab fragments were purified from the supernatant.

### Characterisation of the allergen-binding properties of purified Fab fragments

The allergen-binding specificity of the purified anti-rMal d 1 and anti-rApi g 1 Fab fragments was analysed by competitive ELISA. The immobilisation of the biotinylated allergens onto SA-coated microtiter plate wells was carried out as described earlier. Purified Fab fragments at a final concentration of 15 nM were first incubated with competitor allergen at different concentrations (0.4 nM–14 μM) in solution for an hour at room temperature with rotation. The allergen-Fab fragment complexes were then transferred to the allergen-coated SA-wells. Finally, the amount of bound Fab fragments was detected using alkaline phosphatase conjugated goat anti-mouse Ig (H + L) and *p*-nitrophenylphosphate substrate. The absorbance values were measured at 405 nm.

Due to known similarity in structures and sequences between Mal d 1, Api g 1 and Bet v 1, the binding of rMal d 1 and rApi g 1 -specific Fab fragments against rBet v 1 was also analysed by ELISA. First, a small scale production in *E. coli* RV308 and ELISA with supernatant samples were carried out as earlier. Then, the binding properties were further analysed by ELISA using purified Fab fragments. The biotinylated allergens were immobilised onto SA-coated microtiter plate wells using BSA-Bio as a negative control. Purified anti-rMal d 1 and anti-rApi g 1 Fab fragments were allowed to bind to immobilised allergens at different concentrations (0.03 nM–0.3 μM) for an hour at room temperature with agitation. The detection of the Fab fragment binding was carried out as described previously.

Surface Plasmon Resonance (SPR) experiments of the purified rMal d 1 and rApi g 1 -specific Fab fragments were conducted with BIAcore (T200) at 25°C. Briefly, seven serially diluted concentrations of MA1, MB3, AH7 or AA11 Fab fragments (0–400 nM) were injected at a flow of 30 μl/min to Sensor Chip CAP coupled with biotin capture reagent (GE Healthcare) and biotinylated recombinant allergen. The binding of MA1 and MB3 Fab fragments against rMal d 1 and rBet v 1 as well as AH7 and AA11 Fab fragments against rApi g 1 and rBet v 1 was analysed. As a reference, Fab fragment binding to Sensor Chip CAP with capture reagent was studied. The results were analysed with the Evaluation T200 -software with a 1:1 Langmuir binding model.

The ability of the purified Fab fragments to recognise the apple and celery allergen from natural sources, i.e. apple and celeriac, respectively, were studied with the immunoprecipitation assay. Apple and celeriac extracts were prepared from the locally purchased Golden Delicious apples and raw celery root (celeriac) according to the method of Björkstén *et al*. [50]. Briefly, small pieces of apple and celeriac were frozen with liquid nitrogen and homogenised prior to suspension into 10 mM potassium phosphate buffer (pH 7.0) containing 2% suspended polyvinylpolypyrrolidone (PVPP, Sigma Aldrich), 2 mM of EDTA (Sigma Aldrich) and 10 mM of sodium diethyldithiocarbamate (DIECA, Sigma Aldrich). The homogenate was stirred vigorously for four hours at 4°C and centrifuged (40 000 g, 30 min, 18°C). The supernatant was passed through a polyvinylidene difluoride filter with a pore diameter 0.45 μm (Millex) and dialysed against 10 mM potassium phosphate buffer (pH 7.0) for 18 h at 4°C. The allergen extractions were confirmed by immunoblot. In the immunoprecipitation assay, both allergen extract and recombinant allergens (Biomay) were used. The allergen-specific Fab fragments were immobilised onto protein G beads (Dynabeads, Dynal) according to the manufacturer’s instructions (25 μg protein/ 25 μl beads). Allergen samples were incubated with protein G-Fab fragment beads for two hours at room temperature with rotation. Allergen-Fab fragment complexes were eluted with 0.1 M glycine-HCl (pH 2.0) and immediately neutralised with 1 M Tris buffer. The eluted samples were analysed on silver-stained SDS-PAGE.

The usability of allergen-specific Fab fragments for the detection of allergens in sandwich ELISA was studied. In the sandwich ELISA, the immobilised antibody was the purified Fab fragment (MB3 Fab fragment in the case of Mal d 1 and AH7 Fab fragment in the case of Api g 1) whereas the detection antibody was displayed on the tip of the phage particle (MA1 Fab fragment displaying phage in the case of Mal d 1 and AA11 Fab fragment displaying phage in the case of Api g 1). The M13 bacteriophages displaying Fab fragments were prepared as previously. The purified Fab fragments (1 μg) were immobilised passively on Maxisorp plate wells (Nunc). After immobilisation, the wells were blocked with PBS containing 0.5% BSA for an hour at room temperature with agitation. Then, the allergen (Biomay, 1 μg) was added followed by the addition of the phage particles displaying the other Fab fragment specific to the allergen at a concentration of 10^10^ cfu/ml in PBS containing 0.05% Tween. The bound phage particles were detected using anti-M13-HRP conjugate and ABTS as previously and the absorbance values were measured at 405 nm. As controls, the absorbance of Fab fragment, phage particles (10^10^ cfu/ml) displaying the Fab fragment and helper phage particles (10^11^ cfu/ml) were analysed.
